# Generation of cortical neurons through large-scale expanding neuroepithelial stem cell from human pluripotent stem cells

**DOI:** 10.1186/s13287-020-01939-6

**Published:** 2020-10-02

**Authors:** Shumei Zhao, Kui Duan, Zongyong Ai, Baohua Niu, Yanying Chen, Ruize Kong, Tianqing Li

**Affiliations:** 1 Yunnan Key Laboratory of Primate Biomedical Research, Institute of Primate Translational Medicine, Kunming University of Science and Technology, Kunming, China; 2 Xi’an ChaoYue Stem Cell Co, Ltd, Xi’an, China

**Keywords:** Human pluripotent stem cells, Neuroepithelial stem cells, Large-scale suspension culture, Cortical neurons

## Abstract

**Background:**

Considerable progress has been made in converting human pluripotent stem cells (hPSCs) into cortical neurons for disease modeling and regenerative medicine. However, these procedures are hard to provide sufficient cells for their applications. Using a combination of small-molecules and growth factors, we previously identified one condition which can rapidly induce hPSCs into neuroepithelial stem cells (NESCs). Here, we developed a scalable suspension culture system, which largely yields high-quality NESC-spheres and subsequent cortical neurons.

**Methods:**

The NESC medium was first optimized, and the suspension culture system was then enlarged from plates to stirred bioreactors for large-scale production of NESC-spheres by a stirring speed of 60 rpm. During the expansion, the quality of NESC-spheres was evaluated. The differentiation potential of NESC-spheres into cortical neurons was demonstrated by removing bFGF and two pathway inhibitors from the NESC medium. Cellular immunofluorescence staining, global transcriptome, and single-cell RNA sequencing analysis were used to identify the characteristics, identities, purities, or homogeneities of NESC-spheres or their differentiated cells, respectively.

**Results:**

The optimized culture system is more conducive to large-scale suspension production of NESCs. These largely expanded NESC-spheres maintain unlimited self-renewal ability and NESC state by retaining their uniform sizes, high cell vitalities, and robust expansion abilities. After long-term expansion, NESC-spheres preserve high purity, homogeneity, and normal diploid karyotype. These expanded NESC-spheres on a large scale have strong differentiation potential and effectively produce mature cortical neurons.

**Conclusions:**

We developed a serum-free, defined, and low-cost culture system for large-scale expansion of NESCs in stirred suspension bioreactors. The stable and controllable 3D system supports long-term expansion of high-quality and homogeneous NESC-spheres. These NESC-spheres can be used to efficiently give rise to cortical neurons for cell therapy, disease modeling, and drug screening in future.

## Background

The cortex is a target for many disorders of the brain at all stages of life. For example, perturbation of cortical development can lead to neurodevelopmental disorders, such as autism spectrum disorders, while the adult cortex is a major site for certain neurodegenerative and injury diseases including Alzheimer’s disease, frontotemporal dementia, stoke, and cortex injury. Currently, there is still lack of any effective treatment for these diseases. Due to the poor/limited plasticity of adult central nervous system (CNS), it is difficult to produce new neurons by activating endogenous neural stem cells (NSCs) to replace lost neurons [1–3]. Previous reports have shown that NSCs transplantation have a therapeutic effect on neurological diseases [4, 5] and even reconstruct the damaged neural network [6–10]. At present, several suspension culture systems of NPCs or NSCs have been established [11–14], but they cannot stably and reproducibly provide high-quality stem cells, such that they are difficult to meet the requirements of cell quality and quantity for cell transplantation. In addition, the differentiation potential of human pluripotent stem cells (hPSCs) in vitro also provides a unique opportunity to study normal and abnormal corticogenesis.

The cerebral cortex contains dozens of neuron subtypes and is the most complex structure in the mammalian brain. PSCs have been successfully induced into mature brain excitatory projection neurons by adding some small molecule compounds [15–17]. To simulate the development of the brain and the cortex, PSCs were formed into mini brain-like organoids by 3D suspension culture [18–20]. However, the limitations of these methods are poor controllability and difficulty in obtaining cortical cells in large quantities, which hinders their clinical applications in regenerative medicine. Although different systems have been established to directly induce iPSCs into neurons or cortical spheroids in the stirred suspension bioreactor, these neurons obtained by these methods lack of proliferation capabilities and cannot be passaged [21, 22]. Thus, the conventional adherent culture and direct cortical differentiation from hPSCs pose challenges to mass production of high-quality cortical neurons and hamper the routine application of hPSC-derived lineages in the disease modeling and regenerative medicine.

During the development of mammalian brain, NSCs mainly go through two different developmental stages: a neuroepithelial stem cell (NESC) stage and a radial glial progenitor cell (RGPC) stage [23]. RGPCs are heterogeneous and have limited neurogenic differentiation [24, 25]. In contrast, NESCs are NSCs locating at the early-formed neural tube stage with a strong self-renewal ability and differentiation potential and can extensively generate cortical neurons [26, 27]. These characteristics endow the NESCs as ideal donor cells for potential therapeutic applications in repairing lost cortical neurons. Using a combinatorial small molecule and growth factors, we previously identified one condition to rapidly differentiate hPSCs into NESCs [26, 28]. However, the large-scale suspension culture of NESCs has not been established, and the technology about the large-scale production of NESCs is still challenging. In addition, whether these NESCs could massively produce cortical neurons is unclear. In the study, we developed a scalable suspension-culture system to largely yield high-quality NESC-spheres. The suspension-culture system supported enlargement of NESC-spheres from plates or dishes to stirred bioreactors. Moreover, these NESC-spheres efficiently produce mature cortical neurons.

## Materials and methods

### hESC culture

Human embryonic stem cell lines hESC1 and BG02 were maintained on MEFs as previously described [29]. Among them, hESC1 is an embryonic stem cell line developed by our laboratory [30]. BG02 cell line was gifted from Pro. Zheng Lab of Kunming Institute of Zoology, Chinese Academy of Sciences. Human pluripotent stem cells H9 are suspension cultured in the AIC medium without feeders or matrigel [30]. The AIC medium [30] consisted of modified N2B27 medium supplemented with 10 ng/ml Activin A (Peprotech, 120-14E), 2 μM IWP-2 (Selleck, S7085), and 0.6 μM CHIR99021 (Selleck, S2924).

### Induce NESC production from hESCs

hESCs were digested into small clumps for suspension culture on ultra-low attachment plates (Corning, 3471) in the NESC-derived medium [26], which is composed of Advance DMEM/F12 (Gibco, 10565-018): Neurobasal media (Gibco, 21103-049) (1:1) supplemented with 1% N2 (Gibco, 17502-048), 2% B27 (Gibco, 17504-044), 1% Glutmax (Gibco, 35050-061), 10 ng/mL OsrbFGF (*Oryza sativa* recombinant human basic fibroblast growth factor, Wuhan Healthgen, China, HYC005M01), 3 μM CHIR99021 (Selleck, S2924), 5 μM SB431542 (Cellagen technology, C7243), 0.2 μM Compound E (Calbiochem, 565790), 0.1 μM LDN193189 (Selleck, S2618), and 0.1 mM β-mercaptoethanol (Sigma, M3148). After suspension culture for 6 days, neuron bodies (NBs) were digested into single cells and inoculated into ultra-low attachment plates with CHbFSB+LIF culture medium. The CHbFSB+LIF culture medium [26, 28, 31] is composed of Neurobasal medium, 1% N2, 2% B27, 1% NEAA (Gibco, 11140-050), 1% Glutmax, 3 μM CHIR99021, 5 μM SB431542, and 10 ng/ml OsrbFGF surplus with 1000 U/ml hLIF (Millipore, LIF1050).

### Suspension and long-term expansion of hNESC-spheres

To extensively expand NESCs in vitro, NESCs were digested into single cells and cultured in ultra-low attachment plates. They were cultured in chemically defined CHbFSB+LIF or CHbFSB culture medium. The CHbFSB culture medium consists of Neurobasal media surplus with 0.25% N2, 0.5% B27, 1% NEAA, 1% Glutmax, 3 μM CHIR99021, 5 μM SB431542, and 10 ng/ml OsrbFGF. TrypLE™ Express Enzyme (Gibco, 12,605,028) was diluted for 2 times with PBS (Sigma, D5652) to digest NESCs for encouraging cell propagation when passaging. NESCs were routinely passaged at 1:3 to 1:4 ratios every 3 days.

### Large-scale expansion of hNESC-spheres

Digested hNESCs (passage 19) were inoculated into a 125 ml suspension bioreactor (Wiggens, BIOMIX Control MS4) with a 100-ml CHbFSB medium at the cell density of 3 × 10^5^ cells/ml. Every 3 days, the NESC-spheres were dissociated and passaged using TrypLE™ Express Enzyme: PBS (1:2). The agitation rate of NESCs growing in a stirred suspension bioreactor is 60 rpm. The bioreactor was housed in a humidified incubator with 5% CO2 at 37 °C. The NESCs were fed 2 days after inoculation by replacing 50% of the medium with the fresh medium.

### Transcriptome analysis

Total RNA was isolated from NESC-spheres cultured in the CHbFSB+LIF or CHbFSB medium using the RNeasy Mini Kit (QIAGEN, 74106). RNA sequencing libraries were constructed using the NEBNext® Ultra RNA Library Prep Kit for Illumina® (NEB England BioLabs, E7530L). The fragmented and randomly primed 2 × 150-bp paired end libraries were sequenced using an Illumina HiSeq X Ten. The generated sequencing reads were mapped against human genome build hg38 using HISAT2 alignment software tools. The read counts for each gene had calculated and normalized with StringTie software [32]. For subsequent analysis of gene expression, genes were retained in both datasets if they were expressed in at least one sample with an FPKM > 5 threshold. Heat maps were generated using pheatmap package in the R software (https://www.r-project.org/).

### 10x single-cell gene expression analysis

We performed RNA amplifcation of single cell from hNESC-spheres with the 10X Genomics plaform. Nine thousand seven hundred sixty-nine single cells were sequenced with the Illumina NextSeq 500. The raw data were first analyzed by Cellranger. The output of the Cellranger (v3.1) was used to create a Seurat object with Seurat packages (v3.0) [33]. The UMAP non-linear dimensional reduction techniques were used to visualize and cluster analysis based on the same PCs input. The VlnPlot tool was used to show marker gene expression probability distributions across clusters. The FeaturePlot tool was used to visualize feature expression on UMAP plots. The differentially expressed genes between two specific groups of cells have been performed with FindMarkers function based on the non-parameteric Wilcoxon rank sum test.

### Cortical neuron differentiation of hNESC-spheres

To induce differentiation, hNESC-spheres were digested into single cells and cultured on plates coated with laminin (5 μg/ml) and poly-ornithine (Sigma, 15 μl/well) in the differentiation medium. The differentiation medium [26] was composed of Neurobasal, 0.5% N2, 1% B27, 1% NEAA, and 1% Glutmax. The medium was replaced by the fresh medium every 3 days. On day 6 post-differentiation (pdD6), 10 ng/ml BDNF (Gibco, PHC7074) and 10 ng/ml GDNF (Gibco, PHC7044) were added into the medium to induce terminal maturation of neurons. To get more mature neurons, 2 × 10^3^/cm^2^ mouse astrocytes were co-cultured with the differentiated neurons at pdD6. Mouse astrocytes were isolated from new-born mouse cortex. Before used for co-culture with neurons, astrocytes were cultured for four passages to exclude the contamination of neurons.

### Immunocytochemistry

Cells were fixed with 4% paraformaldehyde for 20 min, washed with PBS for three times, treated with 0.2% Triton X-100 (sigma, X100) for 30 min, washed with PBS for three times, incubated in blocking buffer (3% BSA (Generay, 9048-46-8) in PBS) for 30 min at room temperature, and washed with PBS for three times. The cells were then incubated with primary antibody overnight at 4 °C (Table S1). The cells were washed with PBS including 0.05% Tween 20 for three times and incubated with Alexa 488, 568, or 647 Fluor-conjugated secondary antibodies (Thermo Fisher: goat anti-rabbit, A31573; goat anti-mouse, A10037; donkey-anti-goat, A11055; Jackson ImmunoResearch: donkey anti-chicken, 703-545-105; 1: 600) for 2 h at room temperature. Nuclei were visualized by DAPI staining (Sigma, 32,670).

### Cryosections of hNESC-spheres

hNESC-spheres from different generations (passages 5, 15, 25) were harvested at the day 3 of culture. hNESC-spheres were fixed with 4% paraformaldehyde for 30 min and washed with PBS for three times. They were dehydrated by adding 20% sucrose for 10 min, embedded in O.C.T (Optimum Cutting Temperature Compound, Sakura, 4583), and frozen at − 20 °C. These samples were cut into 10-μm-thick slices and then subjected to immunofluorescence staining.

### Statistical analysis

All of experiments including immunocytochemistry were at least performed repeated 3 times. Image J and GraphPad prism software were used to measure the diameters of NESC-spheres over culture. Quantification data were represented as mean ± standard deviation (SD). Comparison of the outcome of variables in various experiments was assessed by unpaired Student’s *t* test. *P* value of < 0.05 was considered as significance.

## Results

### Establishing a suspension culture system of human NESCs

**Fig. 1:**
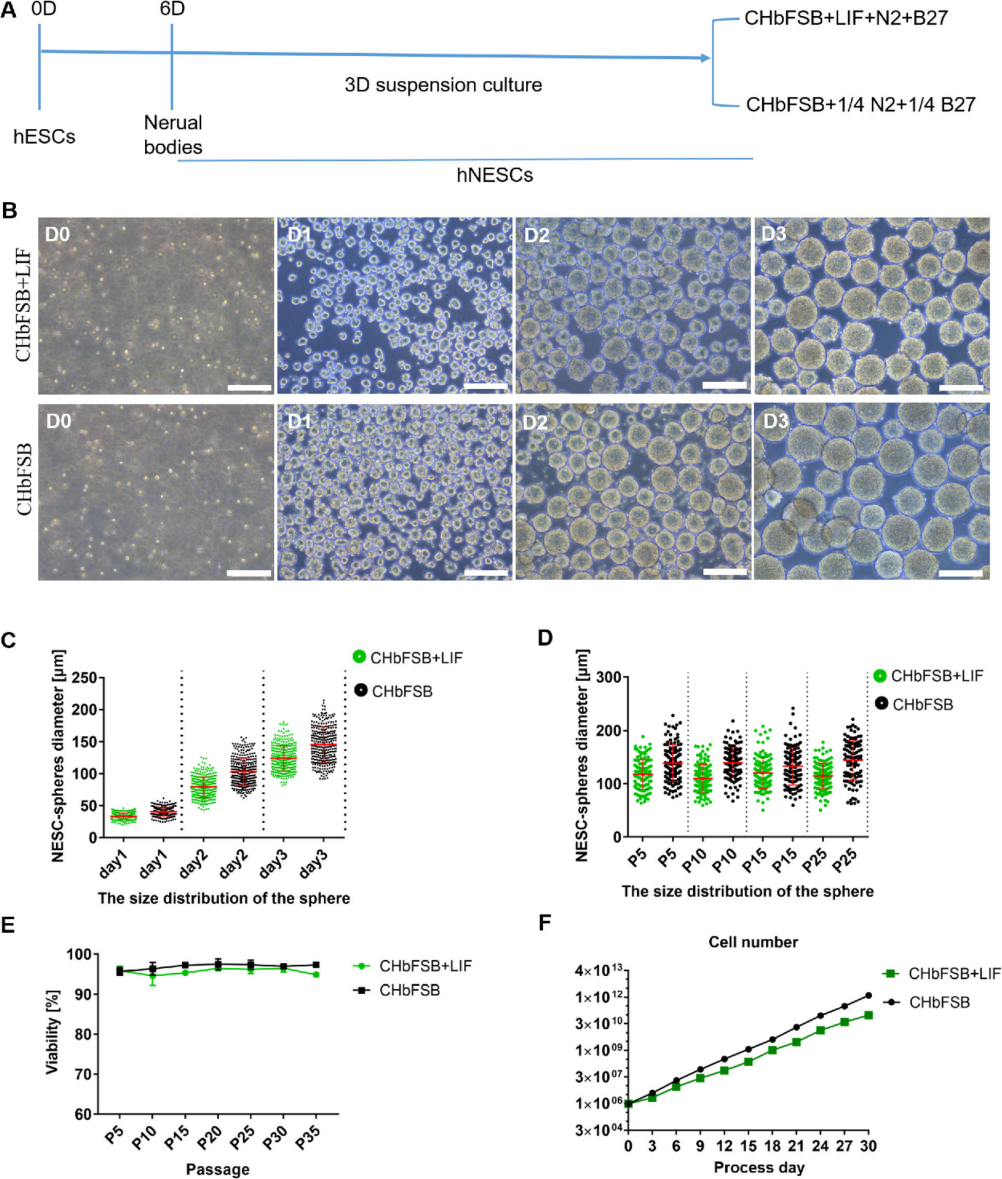
The CHbFSB+LIF and CHbFSB support suspension expansion of NESCs. **a** Schematic representation of the induction and long-term suspension expansion of NESCs. **b** Phase-contrast of NESC-spheres from day 0 to day 3 in the CHbFSB+LIF and CHbFSB media, respectively. **c** The diameter dynamics of NESC-spheres over culture. Each dot visually represents a sphere. **d** The diameters of NESC-spheres during serial passages. **e** Viability dynamics of different passaged NESC-spheres (*n* = 3 independent experiments). Data are represented as mean ± SD. **f** The growth curve of NESCs growing in the CHbFSB+LIF and CHbFSB, displaying the prospect of exponential growth over serial passages. Scale bars, 200 μm. D, day; P, passage

Following our previous protocol (Fig. 1a) [26, 28], we successfully induced hESCs into highly enriched NESCs using a cocktail including bFGF, CHIR99021 (a GSK3 inhibitor), SB431542 (a transforming growth factor β inhibitor), Compound E (a Notch inhibitor), and LDN193189 (an inhibitor of ALK2 and ALK3) [34–37]. Using this system, hPSCs in suspension culture in the AIC medium [30] were also successfully induced into NESCs (Fig. S3A-C). Considering that suspension-culture is a key to achieve a large-scale production of cells, we sought to develop a suitable suspension-culture system of NESCs. Our previous works showed that the CHbFSB+LIF medium (including bFGF, LIF, CHIR99021, and SB431542 supplemented with 1% N2 and 2% B27) allows clonal expansion of hESC-derived NESCs to develop into miniature neural tube (NT)-like structures in the adherent culture [26, 28]. In our initial suspension-culture, NESCs at post-differentiation day 6 (pdD6) were digested into single cells and subjected to 3D suspension propagation in the CHbFSB+LIF medium. As expected, single hNESCs grown in the CHbFSB+LIF quickly formed NESC-spheres at day 1 and exhibited strong proliferation in the next 2 days (Fig. 1b).

Given that N2 and B27 supplements are costly and usually used to culture differentiated neurons, we guessed that the low concentration of N2 and B27 may be beneficial to NESCs expansion. Based on the recommended concentration of the manufacturer, we diluted the N2 and B27 four times, respectively. An our previous study together with another report have showed that the removal of leukemia inhibitory factor (LIF) from the medium cannot affect the human NESCs or NSCs survival and self-renewal [28, 38], suggesting that self-renewal of NESC-spheres at high cell density may not require exogenous LIF. Thus, we designed the CHbFSB medium (including bFGF, CHIR99021, and SB431542 as well as 0.25% N2 and 0.5% B27 supplements) as an optimized medium. Next, we evaluated the expansion of NESC-spheres in the CHbFSB medium. Interestingly, the CHbFSB medium could efficiently support suspension propagation of NESC-spheres (Fig. 1b–f). At day 3, these NESC-spheres were subjected for passaging every 3 days and routinely passaged at 1:4 to 1:6 with a cell density of 3.0 × 10^5^ per milliliter. The NESC-spheres displayed round or ellipsoid morphologies and maintained uniform size and high viability over passaging (Fig. 1c–e).

Next, we compared the growth of NESC-spheres in the two systems. Quantification showed that the diameters of NESC-spheres at day 3 were approximately 124.05 ± 20.00 μm for the CHbFSB+LIF and 146.10 ± 28.34 μm for the CHbFSB, respectively (Fig. 1c). Long-term evaluation showed that uniform sizes of NESC-spheres were stably maintained in the two culture systems over passaging (Fig. 1d). Above 95% of cell viability in NESC-spheres was retained in the two culture systems (Fig. 1e). Cell yield was up to 3~5-fold (CHbFSB+LIF) or 4~6-fold (CHbFSB) every passage (3 days). These cells showed exponential growth over serial passages, resulting in the increase to 1.07 × 10^11^ cells (CHbFSB+LIF) versus 1.39 × 10^12^ cells (CHbFSB) within 30 days without losing obvious proliferative capacity, respectively (Fig. 1f). These results showed that the optimized medium better promoted the proliferation of NESCs, but had no significant (*p* > 0.05) effect on the cell viability of NESCs.

### Human NESC-spheres maintained NESC identity during long-term expansion

**Fig. 2:**
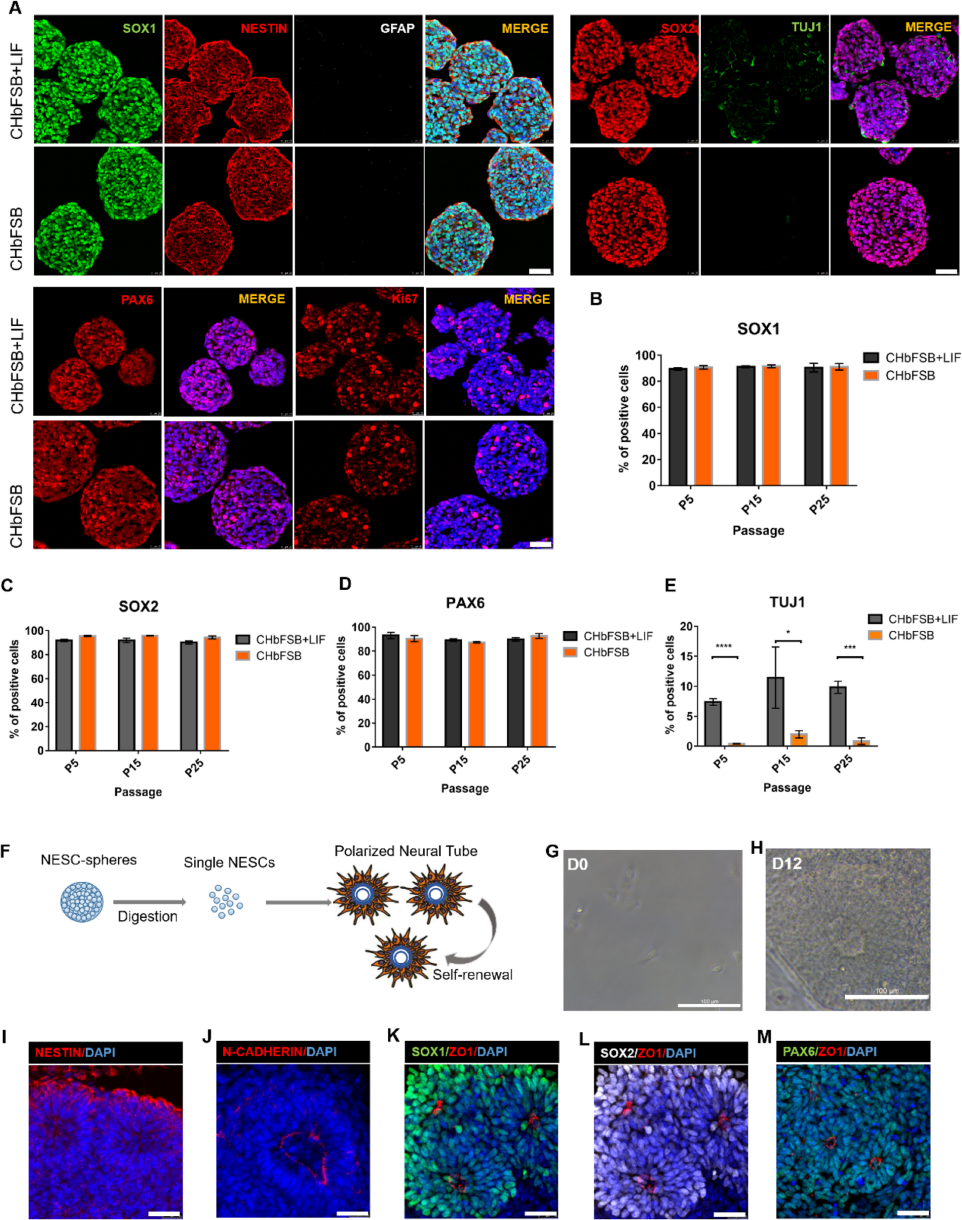
Long-term expanded NESC-spheres maintained NESC characteristics. **a** Immunofluorescence staining of cryosections showed that NESC-spheres from passage 5 at day 3 express NESC markers, such as SOX1, NESTIN, SOX2, and PAX6, and the proliferation-associated marker Ki-67. In contrast, NESC-spheres are negative for GFAP and express TUJ1 in a small number of cells. **b**–**d** Quantification of the number of SOX1 (**b**)-, SOX2 (**c**)-, and PAX6 (**d**)-positive cells in the passage 5, 15, 25 NESC-spheres, respectively. **e** Quantification of differentiated TUJ1-positive neurons in the passaged 5, 15, 25 NESC-spheres, respectively. **f** Schematic representation of single NESCs from spheres self-organization into neural tube (NT)-like structures in the colony assays. **g** Single NESCs in one well of the 96-well plate at day 0. **h** Representative NT-like structures of NESCs at day12. **i**–**m** The polarized NTs express NESC markers, such as NESTIN, N-CADHERIN, SOX1, SOX2, PAX6, and ZO-1. **b**–**e** Data are represented as mean ± SD (*n* = 3 independent experiments). *p < 0.05, ***p < 0.001, ****p < 0.0001 by Student’s *t* test. Scale bars: **g**, **h** 100 μm; others, 50 μm

NESCs generally exist in the embryonic neural plate and neural tube and have strong ability to divide and proliferate [16, 20]. SOX1 is one of the molecular proteins capable of labeling early NESCs [39, 40]. In addition, NESCs specifically express neural precursor markers (PAX6, SOX2, and NESTIN) and a polar molecular marker tight junction protein (ZO-1). Immunofluorescence for NESC-spheres from different passages showed that NESCs growing in the two systems uniformly expressed PAX6, SOX1, NESTIN, and SOX2, but not glial cell marker GFAP (Figs. 2a, S1A, S2A and S3D). It is noted that the NESC-spheres culturing in the CHbFSB system had fewer differentiated TUJ1^+^ neurons than those in the CHbFSB+LIF system (Fig. 2a, e). However, no significant difference in the expression of SOX1, SOX2, and PAX6 was observed in the NESC-spheres from the two culture systems (Fig. 2b–d and S2B). Together, the CHbFSB system better supported the long-term expansion of NESC-spheres than the CHbFSB+LIF. Therefore, we used the CHbFSB for subsequent experiments unless otherwise noted.

We next explored whether NESCs from spheres maintain the ability to self-organize into NT-like structures by clone formation assays of single cells (Fig. 2f). NESC-spheres were digested into single cells and seeded into 96-well plates (Fig. 2g). NESCs self-organized into NT-like structures at day 12 (Fig. 2h). These NT-like structures uniformly expressed NESTIN, SOX2, SOX1 and PAX6, clustered ZO-1 and N-CADHERIN marking the luminal side (Fig. 2i–m). Together, NESCs after extensive expansion still retained the NESC identity.

### Large-scale expansion of human NESC-spheres

**Fig. 3:**
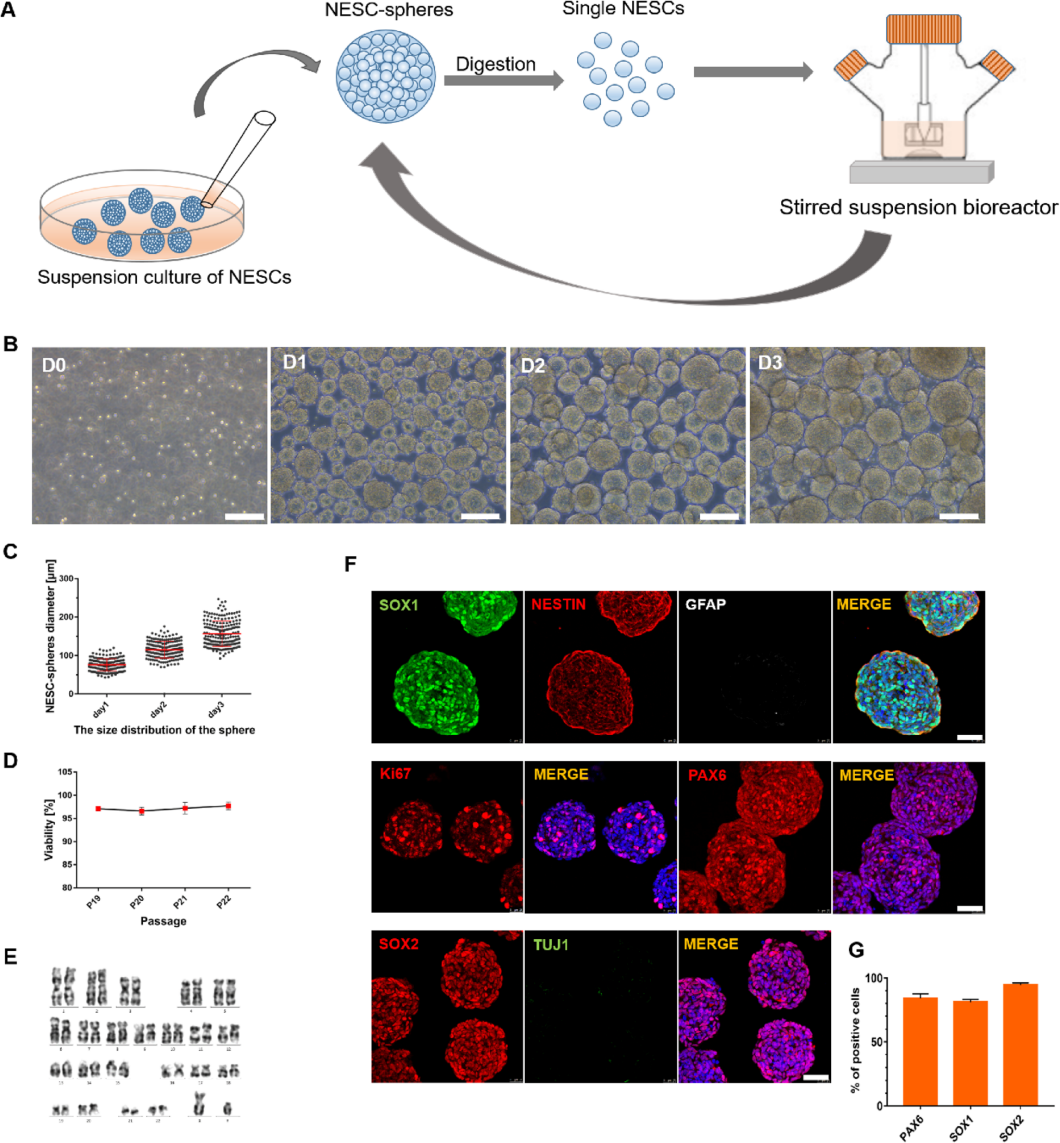
NESC-spheres cultured in a stirred suspension bioreactor. **a** Schematic representation of NESC-spheres cultured in the CHbFSB medium using a stirred suspension bioreactor. **b** Phase-contrast of NESC-spheres from day 0 to 3 in a suspension stirred bioreactor. **c** The diameter dynamics of the NESC-spheres in the suspension stirred bioreactor at the days 1, 2 and 3. Data are represented as mean ± SD (*n* = 3 independent experiments). **d** Viability of different passaged NESC-spheres (*n* = 3). **e** The NESC-spheres maintained normal karyotype after expansion of 23 passages. **f** Immunostainings show that NESC-spheres in the stirred suspension bioreactor express NESC markers, SOX1, NESTIN, SOX2, and PAX6, and the proliferation-associated marker Ki-67. In contrast, NESC-spheres are negative for GFAP and expressed TUJ1 in a small number of cells. **g** Quantification of PAX6, SOX1, and SOX2 positive cells in passage 4 NESC-spheres. Data are represented as mean ± SD (*n* = 3 independent experiments). No significant difference (*p* > 0.05) was found. Scale bars: **b** 200 μm; **f** 50 μm

To test the potential of large-scale culture, NESC-spheres expanded in ultra-low attachment plates were digested into single cell suspensions and inoculated in a 125 ml suspension bioreactor with the stirring rate of 60 rpm (Fig. 3a). As expected, NESCs proliferated normally and formed aggregates in the bioreactor (Fig. 3b). On day 3, average diameters of these NESC-spheres increased up to approximately 156.97 ± 32.78 μm (Fig. 3c) and NESCs held > 95% of cell viability when digested into single cells for passaging (Fig. 3d). This stirring suspension culture facilitated serial passages of NESC-spheres because of easily controlling sphere diameter and minimizing cell apoptosis in the sphere center. In the bioreactor, NESC-spheres maintained extensive proliferation and NESC identity for at least 23 passages and remained a normal diploid karyotype (Fig. 3e). Immunostaining showed that NESC-spheres expressed SOX1, NESTIN, SOX2, and PAX6 (Fig. 3f, g). No expression of GFAP and TUJ1 indicated the absence of glial cell or neuron differentiation in NESC-spheres (Fig. 3f). The wide expression of Ki-67 further revealed the strong proliferation of NESCs (Fig. 3f). The above data showed that the system was suitable for large-scale production of NESCs in stirred suspension bioreactors.

### Molecular and cellular homogeneity of human NESC-spheres

**Fig. 4:**
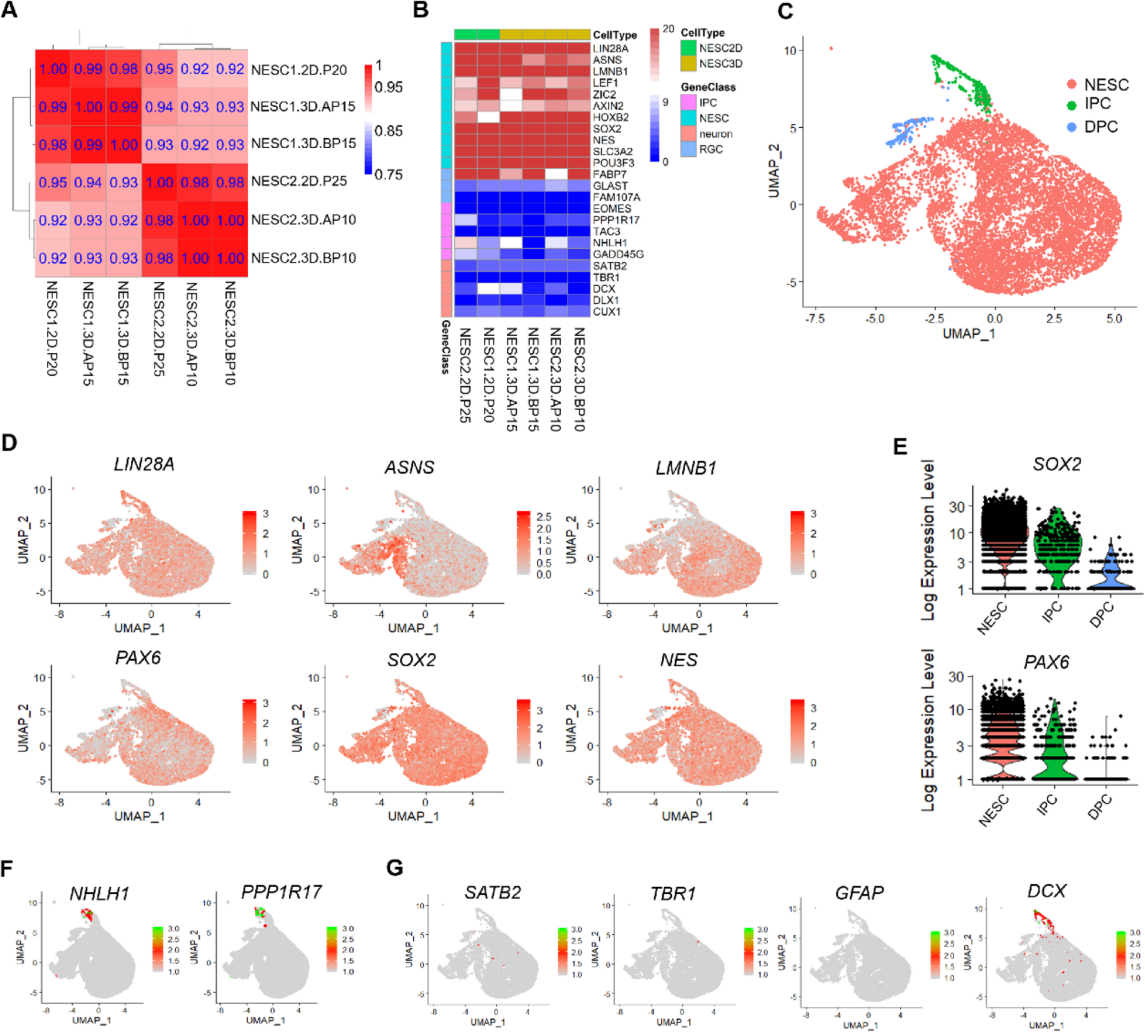
Molecular and cellular homogeneity of NESC-spheres. **a** Sample correlation (Spearman) of transcriptomes among the NESC1-2D-P20, NESC2-2D-P25, NESC1-3D-AP15, NESC2-3D-AP10, NESC1-3D-BP15, and NESC2-3D-BP10. Two cell lines were analyzed and termed as NESC1 (from hESC1) and NESC2 (from BG02). 2D, two-dimensional culture; 3D, three-dimensional suspension-culture; P, passage. A, the CHbFSB+LIF medium; B, the CHbFSB medium. **b** The heatmap of genes relative to NESCs, radial glial cells (RGCs), intermediate progenitor cells (IPCs), and neurons. **c**–**g** A comprehensive transcriptome analysis of single cells using 10x genomics platform. **c** Uniform manifold approximation and projection (UMAP) plots of 9769 cells from NESC-spheres (P28) colored by cluster annotation. Dots, individual cells. Red, NESCs; green, IPCs (intermediate progenitor cells); blue, DPCs (differentiating-prone cells). **d** NESCs are colored according to the expression levels of the indicated markers on the UMAP map (red, high; gray, low). **e** Violin plots show expression levels and distributions of *SOX2* and *PAX6* in different three clusters. **f**, **g** UMAP plots of cells colored by expression of selected marker genes relative to astrocytes, neurons, and IPCs. The different colors indicate the difference of expression level (green, high; gray, low)

To identify NESC-spheres features, the global transcriptomes of hNESC-spheres growing in different culture systems including two-dimensional adherent (2D) and three-dimensional suspension (3D-CHbFSB+LIF and 3D-CHbFSB) were analyzed via RNA sequencing (RNA-seq). Sample correlation (Spearman) showed that the transcriptomes of NESCs between cell lines clustered to closer than that different culture conditions (Fig. 4a). We found that NESC-spheres highly expressed many genes, such as *LIN28A* [41], *ASNS* [42], *LMNB1* [43], *ZIC2* [44], *AXIN2* [45], *LYAR* [46], and *LEF1*, which have been reported to play critical roles in NT development (Fig. 4b). In contrast, genes relative to RGC, IPC, and neuron displayed no or low expression under all culture conditions (Fig. 4b). Together, the transcriptome data confirmed the NESC identity of NESC-spheres over extensive passaging.

To further monitor cell homogeneity after long-term expansion of NESC-spheres, we performed high-throughput single-cell RNA-sequencing (scRNA-seq) for late-passage cell populations using a 10x-genomics platform. We digested the NESC-spheres from passage 28 into single cells, detected 9769 cells with 4425 genes and analyzed their molecular characteristics. The analysis identified 3 distinct cell clusters, a NESC cluster accounting for 94.58% of the total cell population, a DPC (differentiating-prone cell) cluster (1.81%), and an IPC (intermediate progenitor cell) cluster (3.61%), based on their gene expression patterns (Fig. 4c). Cell-type-enriched genes were identified by comparing each cluster. NESCs uniquely expressed NESC genes, such as *LIN28A*, *ASNS*, *LMNB1*, *PAX6*, *SOX2*, and *NES*/*NESTIN* (Fig. 4d). DPCs are found to express *SOX2* but not *PAX6* (Fig. 4e), indicating these cells in the cluster were locating in the differentiating routine. In contrast, IPCs expressed the IPC marker genes *NHLH1* and *PPP1R17* and partially expressed *DCX* (a neuron marker) (Fig. 4f, g). In addition, all cells did not express the astrocyte marker *GFAP* and the cerebral cortex neuron markers *SATB2* and *TBR1* (Fig. 4g). These results demonstrate that cells in largely expanded NESC-spheres are highly homogenous.

### Large-scale expansion of NESC-spheres generated mature cortical neurons

**Fig. 5:**
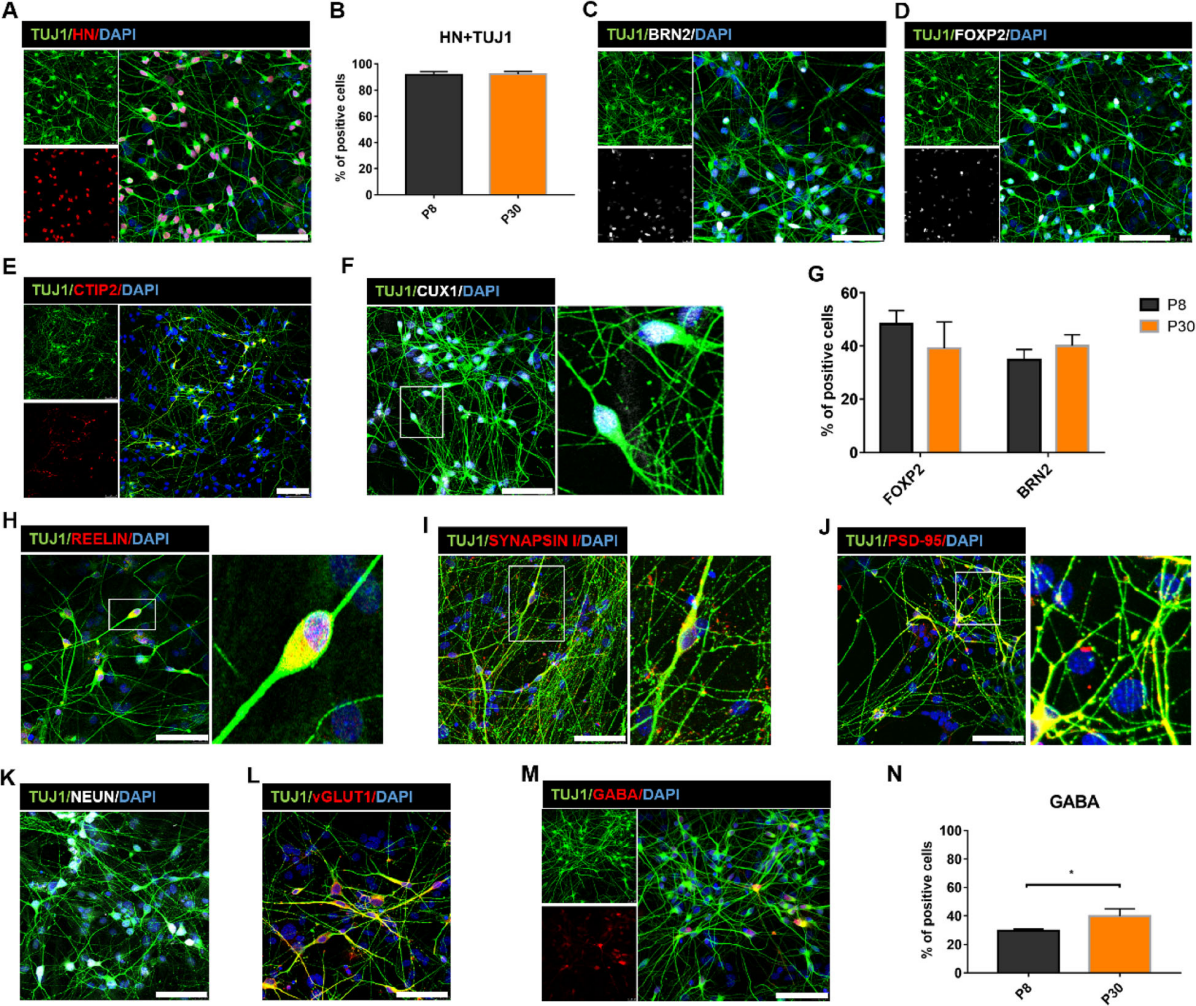
NESC-spheres spontaneously differentiate into cortical neurons. **a** Differentiated neurons co-cultured on mouse astrocytes uniformly express a human-specific marker HN (human nucleus), suggesting that these neurons are from human NESC-spheres but not from mouse. **b** Quantification of NESCs early passage (P8) and late passage (P30) differentiated total neurons. HN^+^ neurons indicate human-derived neurons. **c**, **d** Differentiated BRN2^+^ and FOXP2^+^ cortical neurons from NESC-spheres. **e**, **f** Differentiated CTIP2^+^ and CUX1^+^ cortical neurons from NESC-spheres. **g** Quantification of deeper layers FOXP2^+^ neurons and upper layers BRN2^+^ neurons in early passage (P8) and late passage (P30) differentiation of NESCs. **h** Differentiated REELIN^+^ cortical neurons from NESC-spheres. **i**, **j** Differentiated mature neurons at pdD 89 expressed synapsin I protein and the post-synapse marker PSD-95 with a punctate pattern. **k** At day 34 after post-differentiation (pdD34), differentiated neurons from NESC-spheres express a mature neuron marker NeuN. **l**, **m** vGLUT1^+^ glutamatergic and GABA^+^ GABAergic neurons from NESC-spheres. **n** Quantification of NESCs early passage (P8) and late passage (P30) differentiated GABAergic neurons. Data are represented as mean ± SD (*n* = 3 independent experiments). *p < 0.05 by Student’s *t* test. Scale bars, 100 μm. Blue: DAPI, nuclear stain

In the early stages of brain development, NESCs undergo self-renewal through symmetric division or differentiate into neurons by asymmetric division [47]. The cerebral cortex is composed of six layers in anatomical physiology, and layer-specific neurons are generated by sequence. The first layer, II/III/IV layer (intracortical projection neurons), and V/VI layer (corticofugal projection neurons) are defined into the Cajal-Retzius, upper, and deeper layers, respectively [16, 48, 49]. To generate cortical neurons, NESCs were induced to differentiation by removing bFGF, CHIR99021, and SB431542. At day 6 post-differentiation (pdD6), the mouse astrocytes were added into the dishes and co-cultured with differentiated neurons. At pdD 34, more than 90% of differentiated cells from NESCs co-expressed TUJ1 and human nuclei (HN) (Fig. 5a). Furthermore, long-term suspension-expansion did not destroy the differentiation potentials of NESCs (Fig. 5b). After co-cultured with mouse astrocytes, NESCs differentiated into cortical neurons including the BRN2- and CUX1-positive upper layer neurons (Fig. 5c, f), as well as FOXP2- and CTIP2-positive deeper layer neurons [17, 50] (Fig. 5d, e). Quantification of differentiated cells at pdD42 showed that FOXP2-positive neurons from passage 8 (P8) and passage 30 (P30) NESC-spheres were 48.29 ± 5.03% and 39.17 ± 9.89%, respectively (Fig. 5d, g), while BRN2-positive neurons at pdD42 accounted for 34.82 ± 3.88% and 40.14 ± 4.09%, respectively (Fig. 5c, g). These differentiated neurons expressed TBR1, a marker of cortical projection neurons (Fig. S4A). In addition, we also detected a few REELIN^+^ (a marker of Cajal-Retzius neurons) neurons and SATB2^+^ (a marker of layer V- or upper-layer callosal neurons) neurons (Figs. 5h and S4A).

Synapse genesis is a key step in the formation of neural circuits. To test whether these cortical neurons can form physical synapses in vitro, differentiated neurons were co-cultured with mouse astrocytes. Most of differentiated neurons expressed NeuN at pdD34 (Fig. 5k), pre-synaptic protein SYNAPSIN I and post-synaptic protein PSD-95 in their axons in a punctate pattern at pdD89 (Fig. 5i, j). In the cerebral cortex, the vast number of neurons is mainly made up of two types according to secreted neurotransmitters: more than 80% of glutamic-excitatory (glutamatergic) projection neurons and about 20% of GABA aminobutyric acid-inhibitory (GABAergic) neurons [51]. Subtype identification showed that NESCs spontaneously differentiated into glutamatergic neurons and GABAergic neurons (Fig. 5l, m). Quantification data showed that NESCs at passage 8 and 30 gave rise to 29.74 ± 1.26% and 39.78 ± 5.17% of GABAergic neurons, respectively (Fig. 5n). Moreover, these of GABAergic neurons co-expressed calretinin (CR), calbindin (CB), or parvalbumin (PV) (Fig. S4B-D), confirming the identity of inhibitory interneurons.

## Discussion

Here, we developed a system for large-scale production of NESCs derived from hESCs in a stirred suspension bioreactor. Our NESCs stably maintain long-term and large-scale expansion and efficiently give rise to cortical neurons. These NESC-spheres have unique characteristics: (1) retain above 95% of cell viability and uniform size; (2) have robust expansion ability; (3) express NESC markers, such as PAX6, SOX1, NESTIN, and SOX2; (4) express many NT genes, such as *LIN28A*, *ASNS*, and *LMNB1*, but not glial cell marker *GFAP*; (5) self-organize into miniature NT-like structures and cluster tight junction protein ZO-1 and N-CADHERIN into apical side; (6) display high homogeneity of NESCs in spheres; and (7) give rise to cortical neurons.

Neurosphere culture systems are useful for biological studies of developmental processes [52, 53]. Stirred bioreactor suspension culture is an effective way to expand stem cells for regenerative medicine and drug development [54, 55]. However, the content of NSCs in cultured neurospheres is variable and depends on the stage of culture. Previous studies have reported high content of NSCs shortly after separation, but stem cell purity gradually declines in subsequently expanded subcultures [56, 57]. In the study, we developed the medium for the long-term expansion of NESC-spheres. Single-cell transcriptome data demonstrated that the percentage of NESCs in the expanded spheres after long-term passage was as high as 95%. This system provides controlled, stable, and high-quality donor cells for further differentiation and treatment of neurological diseases.

Over the past few decades, several NPCs or NSCs culture systems have been established [11, 12, 14, 35, 58–60], but these developed methods are difficult to provide a sufficient number of homogeneous NSCs for further differentiation, disease study, or clinical stem cell therapy. We previously established a feasible adherent culture system, including LIF, 1% N2, and 2% B27, of hNESCs [26, 28]. However, LIF is not required for the long-term expansion of NSCs or NESCs [28, 38]. According to manufacturer’s suggestion, N2 supplement is recommended for growth and maturation of post-mitotic neurons and B27 supplement increases neuronal survival, implying that their recommended concentrations may result in the neuron differentiation. As expected, we observed that 1% N2 and 2% B27 increased the TUJ1^+^ neurons production, whereas four-time dilutions decreased the TUJ1^+^ neurons differentiation. The further optimization N27 and B27 concentration may improve the suspension-culture system. Compared with other previous suspension culture methods [12, 13], our large-scale suspension system has three advantages. First, the system is defined and lower costs and enables NESC-spheres to stably maintain self-renewal during the long-term expansion. Second, NESC-spheres display uniform size and cell homogeneity along with the typical NESC identities. Third, NESC-spheres can effectively differentiate into cortical neurons. Based on these unique features, it strongly supports the ability to massively generate cortical neurons for drug screening and disease treatment.

During development, the human cortex is composed of six-layer projection neurons [16, 48, 49]. Adult cerebral cortex is poorly plastic, and it is difficult to produce nerve regeneration once damaged. Many neurological diseases, including epilepsy, autism, schizophrenia, and possibly Alzheimer’s disease, are thought to result, at least in part, from the dysfunction of cortical interneuron [8]. Our scaled-up NESC-spheres have strong differentiation ability. NESC-spheres spontaneously initiated differentiation by removing growth factors and inhibitory molecules, and more than 90% of differentiated cells were neurons, including corticofugal projection neurons (deep-layer) and intracortical projection neurons (upper-layer). Interestingly, we also found that NESC-spheres can differentiate into cortical GABAergic interneurons. Calretinin and parvalbumin subtype neurons are present in both the middle ganglia and caudal ganglionic eminence at mouse E13.5, while calbindin neurons originate from the caudal ganglionic eminence [61–63]. Our experiments also showed that these three subtypes of interneurons could be specified from NESC-spheres, providing an ideal platform to study interneuron development. One of main goals for the modern neuroscience is to restore neurological and cognitive functions after brain damage. NESCs have been regarded as a possible donor cells for nerve grafts to repair damaged neural circuits [64]. We confirmed that adherent cultured NESCs are capable of differentiating into functional neurons and integrating into the host cerebral cortex after grafted into mouse and monkey brains [26, 31]. Long-term continuous observation of the host did not show tumors after transplantation, implying that NESCs as donor cells have obvious effects and safety on stem cell therapy [26, 31]. Thus, the efficient and economically viable hNESC-sphere scale-up system developed by us provides a stable and abundant source of cortical cells for cell therapy. However, subsequent works will be performed to confirm whether NESC-sphere differentiation in vivo can replace endogenous injured nerve cells and reconstruct neural networks.

## Conclusions

In summary, we developed a serum-free, defined, and low-cost culture system for large-scale expansion of NESCs with high quality, purity, and homogeneity in stirred suspension bioreactors. These NESC-spheres stably maintain long-term and large-scale through maintenance of unlimited self-renewal and NESCs state. Importantly, NESC-spheres effectively produce different subtypes of mature cortical neurons. This 3D-system overcomes the barriers of mass production of NESCs and can be used in commercial-level cortical neurons production for cell therapy, disease modeling, and drug screening in future.

## Supplementary information

**Additional file 1.**

## Data Availability

All data generated or analyzed during this study are included in this published article and in supplementary figures.

## Abbreviations

NESCs: Neuroepithelial stem cells
NSCs: Neural stem cells
NPCs: Neural precursor cells
hPSCs: Human pluripotent stem cells
bFGF: Basic fibroblast growth factor
RNA-seq: RNA sequencing
CNS: Central nervous system
2D: Two-dimensional
3D: Three-dimensional
ESCs: Embryonic stem cells
RGPCs: Radial glial progenitor cells
NT: Neural tube
pdD: Post-differentiation day
LIF: Leukemia inhibitory factor
scRNA-seq: Single-cell RNA-sequencing
UMAP: Uniform manifold approximation and projection
DPCs: Differentiation-prone cells
IPCs: Intermediate progenitor cells
