# Supplementary material for: Generation of cortical neurons through large-scale expanding neuroepithelial stem cell from human pluripotent stem cells
Source: Stem Cell Res Ther. 2020 Oct 2;11:431. doi: 10.1186/s13287-020-01939-6 (PMC7532602; doi:10.1186/s13287-020-01939-6)
Supplement: Supplementary file 1 — Additional file 1. [file 13287_2020_1939_MOESM1_ESM.docx]

**Supplementary Information**

**
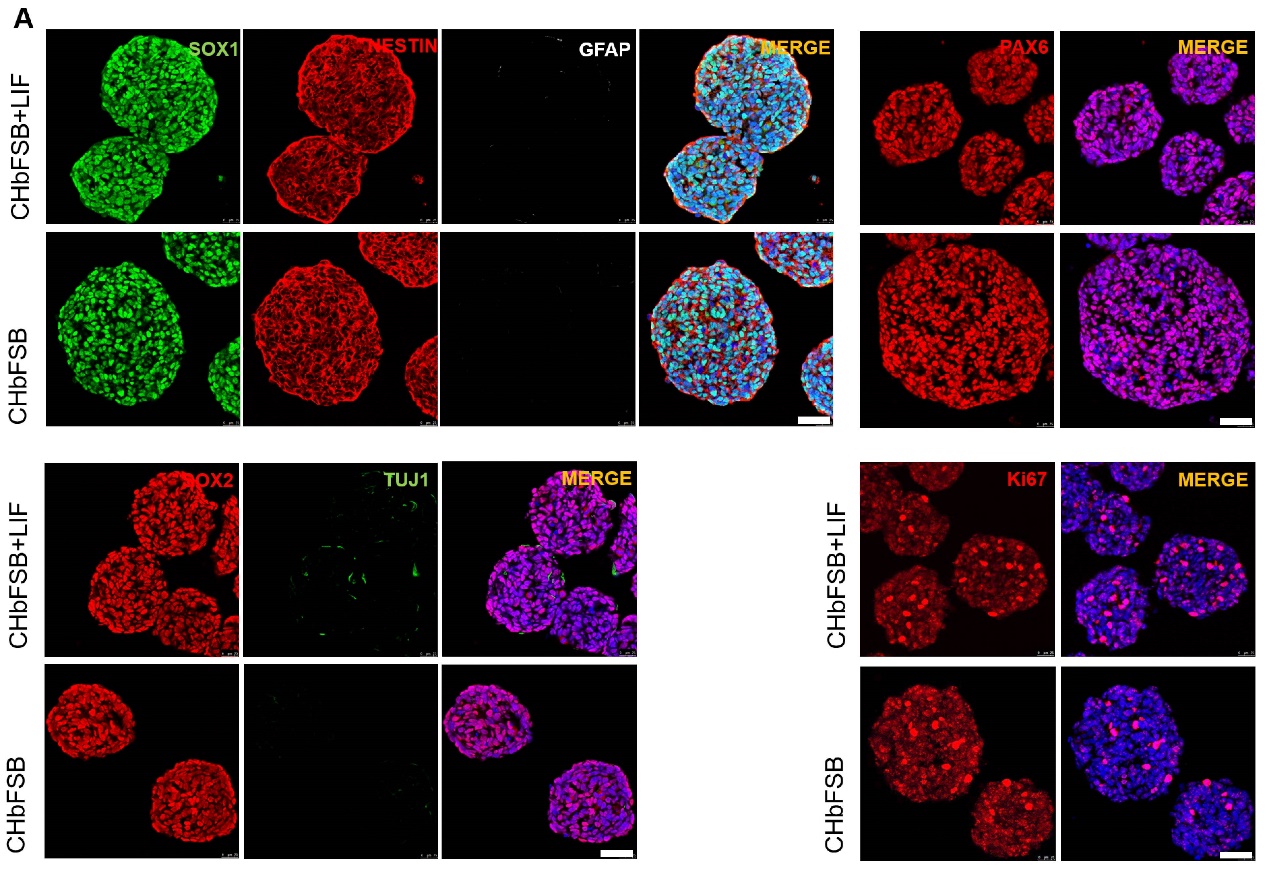
**

Figure S1. The characteristics of NESC-spheres at Passage 25. (A) Immunofluorescence staining of cryosections showed that NESC-spheres (hESC1 line) at the late-passage 25 express NESC markers, SOX1, NESTIN, SOX2 and PAX6, and the proliferation-associated marker Ki-67. However, NESC-spheres are negative for GFAP and express TUJ1 in a small number of cells. Scale bars: 50 μm.

**
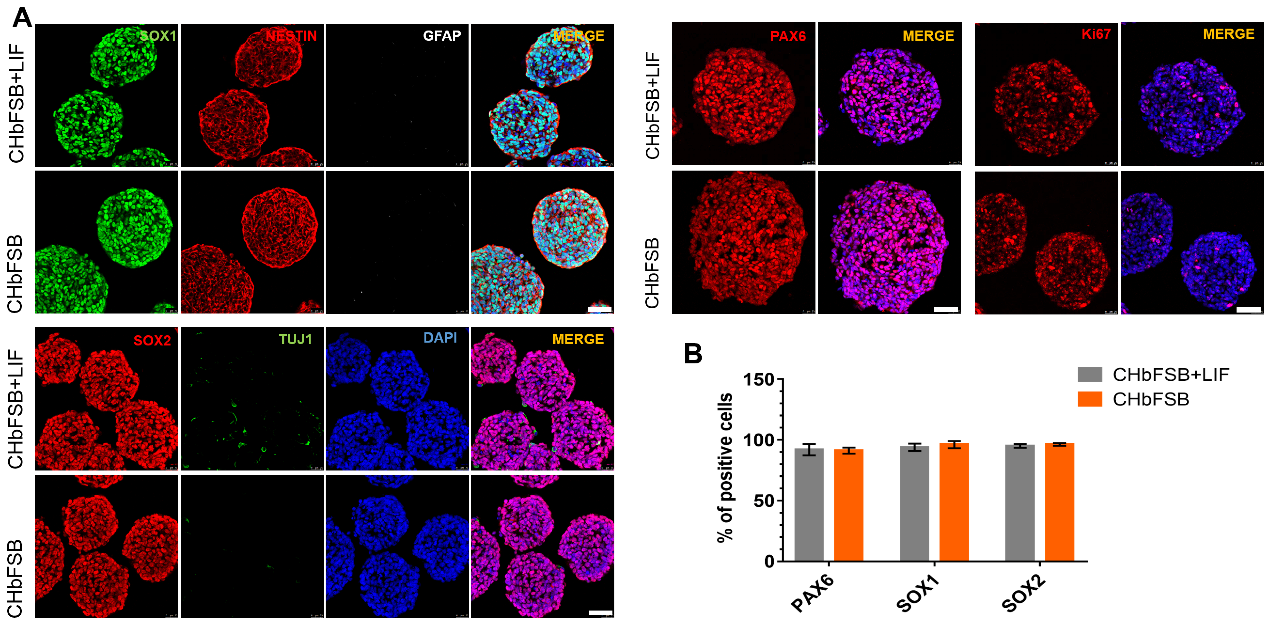
**

Figure S2. NESC-spheres from BG02 cell line maintained NESC characteristics. (A) Immunofluorescence staining of cryosections showed that NESC-spheres express NESC markers, SOX1, NESTIN, SOX2 and PAX6, and the proliferation-associated marker Ki-67, whereas they are negative for GFAP along with TUJ1 expression in a small number of cells. (B) Quantifications of SOX1-, SOX2- and PAX6-positive cells in passage 5 NESC-spheres. Data are represented as mean ± SD (n=3 independent experiments). Scale bars: 50 μm.

**
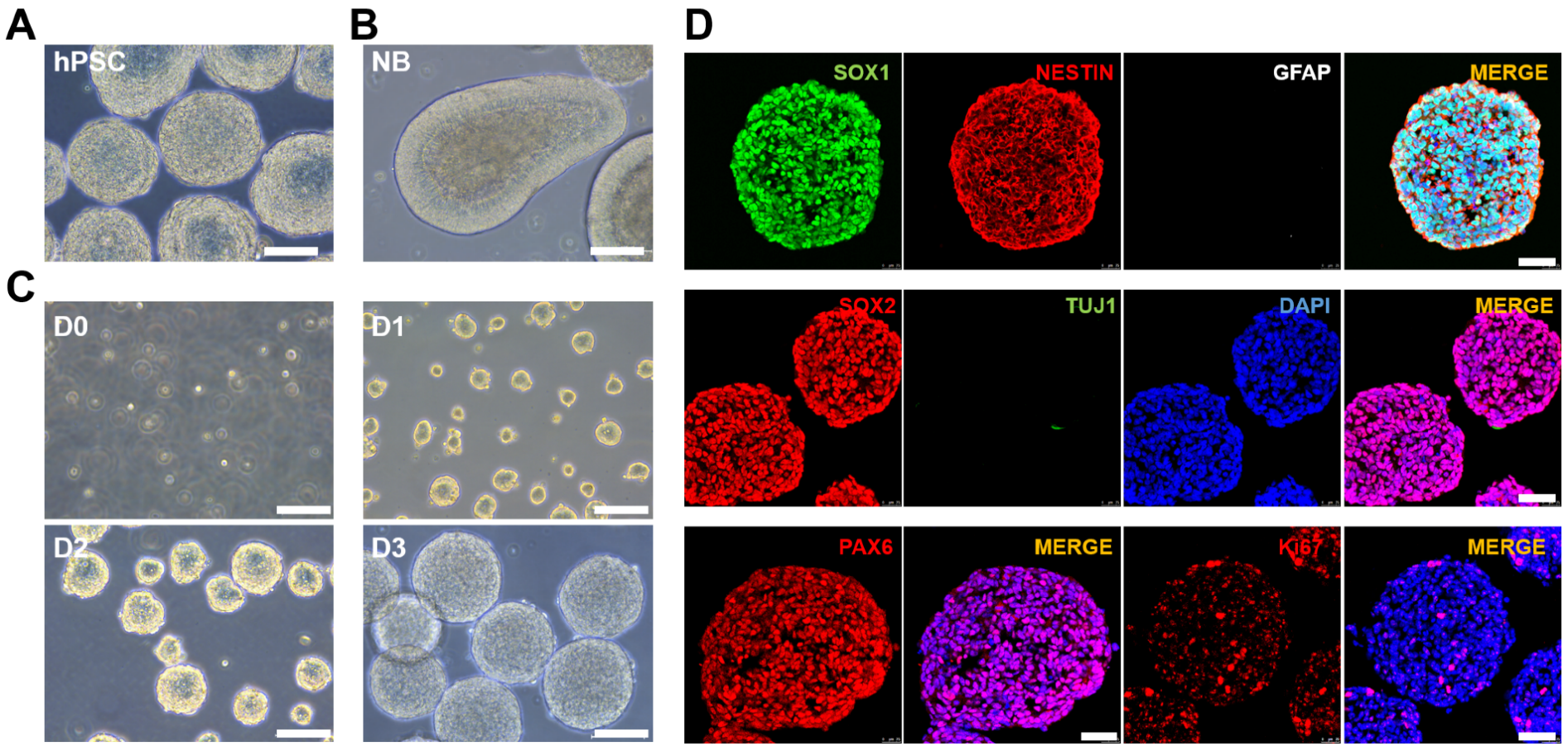
**

Figure S3. NESC-spheres from suspension culture hPSC-H9 cells growing in the AIC medium maintained NESC characteristics. (A) The morphology of suspension culture hPSC-H9 on day 4. (B) Neuron bodies (NBs) cultured in suspension for 6 days formed a two-layer structure. (C) Phase-contrast of NESC-spheres from Day 0 to 3 in the CHbFSB media. (D) Immunofluorescence staining of cryosections showed that NESC-spheres (H9 line) express NESC markers, SOX1, NESTIN, SOX2 and PAX6, and the proliferation-associated marker Ki-67, but are negative for GFAP and TUJ1. Scale bars: A, B and C, 100 μm; others, 50 μm. Abbreviation: D, day.

**
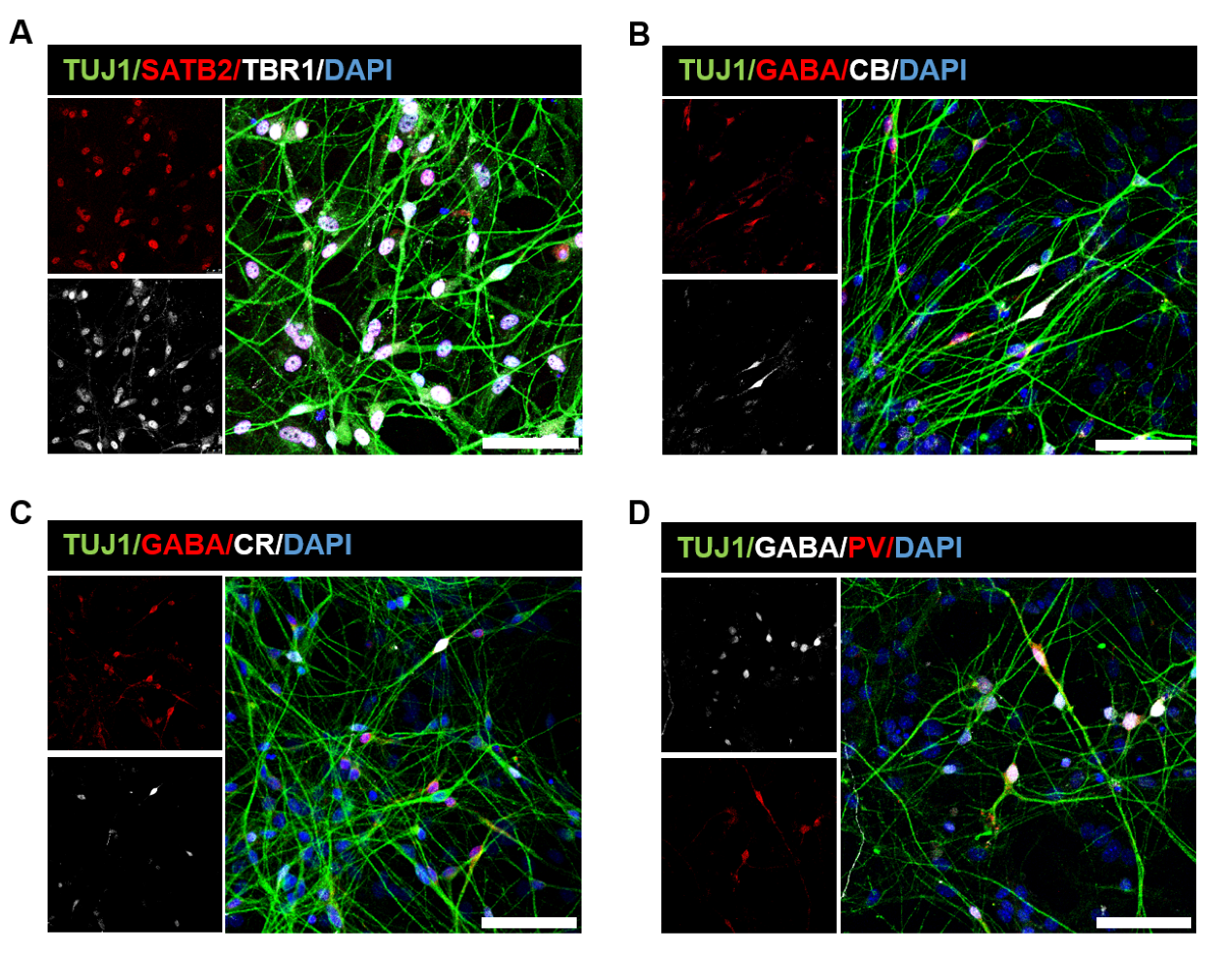
**

Figure S4. NESC-spheres differentiate into cortical neurons and GABAergic neurons. (A) NESC-spheres differentiate into SATB2^+^ and TBR1^+^ cortical neurons at the pdD 42. (B-D) NESC-spheres can differentiate into GABAergic subtype neurons, which co-express Calbindin (CB) (B), Calretinin (CR) (C), and Parvalbumin (PV) (D) at the pdD 62. Scale bars: 100 μm.

Table S1

| Antibodies | Species | Cat No. | Company | Dilution rate |
| --- | --- | --- | --- | --- |
| PAX6 | Rabbit | 901301 | Biolegend Covance | 1:800 |
| SOX1 | Goat | AF3369 | R&D Systems | 1:800 |
| SOX2 | Rabbit | AB5603 | Millipore | 1:600 |
| NESTIN | Mouse | MAB5326 | Millipore | 1:400 |
| Ki-67 | Rabbit | PA5-19462 | Invitrogen | 1:200 |
| ZO-1 | Mouse | 339100 | Invitrogen | 1:50 |
| GFAP | Rabbit | G9269 | Sigma | 1:1000 |
| N-CADHERIN | Rabbit | GTX127345 | GeneTex | 1:200 |
| TUJ1 | Mouse | MAB1637 | Millipore | 1:1000 |
| TUJ1 | Chicken | NB100-1612 | Novus Biologicals | 1:300 |
| Human nuclei | Mouse | MAB1281 | Millipore | 1:500 |
| NeuN | Rabbit | ABN78 | Millipore | 1:500 |
| GABA | Mouse | 015M4831V | Sigma | 1:500 |
| Calretinin | Rabbit | ABN2191 | Millipore | 1:500 |
| Calbindin | Rabbit | AB1778 | Millipore | 1:500 |
| VGLUT 1 | Rabbit | 12331 | CST | 1:200 |
| BRN2 | Rabbit | SAB2501452 | Sigma | 1:800 |
| FOXP2 | Rabbit | ab16046 | ABCAM | 1:800 |
| FOXP2 | Mouse | NBP2-61412 | Novus Biologicals | 1:500 |
| CUX1 | Rabbit | SC13042 | Santa Curz | 1:400 |
| SATB2 | Mouse | ab51502 | ABCAM | 1:200 |
| TBR1 | Rabbit | AB10554 | Millipore | 1:200 |
| Reelin | Mouse | D223-3 | MBL | 1:200 |
| Synapsin I | Rabbit | S193 | Sigma | 1:1000 |
| PSD95 | Mouse | ab2723 | ABCAM | 1:1000 |
| NeuN | Rabbit | ABN78 | Millipore | 1:500 |

Table S1. The primary antibody list.
